# Anterior Cruciate Ligament Injury in Association With Other Knee Injuries in King Abdulaziz University Hospital, Saudi Arabia

**DOI:** 10.7759/cureus.10240

**Published:** 2020-09-04

**Authors:** Ahmad M Tayeb, Abdullah A Almohammadi, Adel H Hegaze, Fawziah Roublah, Khulood A Althakafi

**Affiliations:** 1 Orthopaedic Surgery, College of Medicine, King Abdulaziz University, Jeddah, SAU

**Keywords:** anterior cruciate ligament (acl), meniscal tear, collateral ligament injury, bone injury

## Abstract

Purpose

The purpose of this study was to study the association of anterior cruciate ligament (ACL) injury with meniscal, collateral ligament, and bone injuries using magnetic resonance imaging (MRI) for clinical correlation during ACL repair.

Methods

This was a retrospective cohort study conducted on 136 patients diagnosed with ACL injury by MRI at King Abdulaziz University Hospital (KAUH) between September 2010 and September 2018.

Results

The medial meniscus was injured in approximately half (49.3%) of patients, while the lateral meniscus was injured in 16.2%. Regarding collateral ligament injuries, the medial collateral ligament was injured in six patients (4.4%), the lateral collateral ligament in four patients (2.9%), and both collateral ligaments in three patients (2.2%). There was a significant relationship between the age group and the side of ACL injury (P<0.05) but not between the age group and the presence of an associated injury.

Conclusion

There was no significant relationship between ACL injury and menisci, collateral ligament, or bone injury.

## Introduction

Athletes are at high risk for injuries because they turn on an axis of rotation, turn while decelerating, and repeatedly jump and land. The knee is one of the most commonly injured sites [1]. The knee joint can be divided functionally into two separate joints: the tibiofemoral and the patellofemoral joints [2]. Its articular surfaces are represented by the femoral condyles, which are connected with the corresponding tibial plateau and in between them are the medial and lateral menisci [3]. The stability of the knee is mainly generated by the interaction of the capsule and its ligaments, menisci, muscles, and the cruciate ligaments [3].

The anterior cruciate ligament (ACL) plays an important role as a knee-stabilizing ligament that is frequently injured in athletes and trauma patients [4]. ACL is the major fixating ligament against anterior dislocation of the tibia. ACL is also a minor fixator to valgus rotation [5].

The ACL is one of the most recurrently injured knee ligaments, especially among young athletes [6]. Each year, more than 100,000 new cases of ACL injuries occur (most of these injuries occur as non-contact injuries), and approximately 75,000 ACL reconstructions are performed in the United States [7,8].

The study by Alrubayyi et al. aimed to estimate the prevalence and mechanism of ACL injury among Makkah City, Saudi Arabia. They found that the most common cause of ACL injury was sports (n=181). ACL injuries occurred owing to rapid acceleration (n=18) or deceleration (n=20) as well as traffic accidents (n=16) and work-related causes (n=14) [9].

Another study conducted by Alghamdi et al. found that the prevalence of cruciate ligament injuries among physical education students of Umm Al-Qura University was 5.3%; most injuries involved the ACLs (60%), while 10% involved the posterior cruciate ligaments; the injured ligament was unknown in the remaining 30%. The right knee was involved in 70% of injuries, while 30% were left knee injuries [10].

Injuries to the ACL of the knee are immediately disabling, rehabilitation takes a substantial period of time, and other associated articular injuries are often related to it, as a result, the risk of early-onset posttraumatic osteoarthritis is increased irrespective of the treatment applied [11]. Rotational stability of the knee can be restored by ACL repair; however, it remains unclear whether it prevents knee joint degeneration [12].

Numerous risk factors are associated with an increased risk of ACL injury, such as female sex, bony geometry of the knee, familial predisposition, and prior repair of the ACL [13].

Only a few studies have investigated the association between ACL injury and other associated injuries in the Saudi Arabian population, specifically the western region.

In this study, we addressed the prevalence of ACL injuries and their association with different types of knee injuries among attendees of King Abdulaziz University Hospital (KAUH), Jeddah, Saudi Arabia.

## Materials and methods

Study setting

This study was conducted between September 2010 and September 2018 at KAUH, a tertiary center that is the largest educational hospital on the west coast of Saudi Arabia.

Study design and population

This was a retrospective record review that included all patients between 18 and 40 years old who had a confirmed ACL injury after magnetic resonance imaging (MRI) of the knee between September 2010 and September 2018 at KAUH. All imaging was performed within seven days of the index ACL injury. However, patients with previous knee injuries, congenital abnormalities, or degenerative diseases were excluded. This study was approved by the institutional review board of KAU (Reference No. 359-18). The need for informed consent was waived owing to the retrospective nature of the study.

A total of 136 patients matched our criteria and were included in our study. All patients underwent imaging using a 1.5-T Symphony system (Siemens Medical Solutions, Erlangen, Germany), 3-T Verio system (Siemens Medical Solutions, Erlangen, Germany), or 3-T Skyra system (Siemens Medical Solutions, Erlangen, Germany). All examinations included at least one of the following sequences: axial, coronal, or sagittal images were obtained using conventional, fast spin-echo, turbo spin-echo, proton density-weighted, or T2-weighted sequences. Additional sequences included T1-and T2-weighted gradient-recalled echo sequences. All fast and turbo spin-echo sequences were fat-suppressed.

Data entry and statistical analysis

We used Google Forms, 2016 edition, to develop a data sheet consisting of two sections including the following: demographics (age, sex, and nationality) and MRI findings (type and side of ACL injury, any associated injuries, its relation to meniscus, bone injury, and collateral ligaments). After collecting the data, SPSS version 21 (IBM Corp., Armonk, NY, USA) was used for data entry and data analysis. Categorical variables including primary variables are described using frequencies. Continuous variables for normally distributed are described using mean and standard deviation (SD). Bivariate analysis was conducted for categorical variables using the chi-square test to check for all possible relations of ACL injury. A test with a P-value < 0.05 was considered statistically significant.

## Results

**Figure 1:**
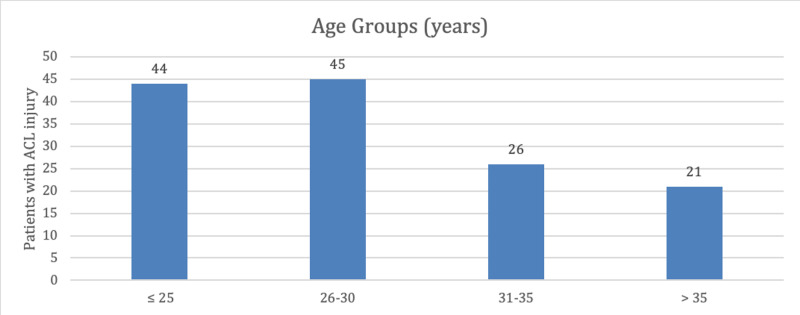
Age groups of the included patients with anterior cruciate ligament (ACL) injury

The age of all included patients with ACL injury ranged from 18 to 40 years, with a mean age of 28.89 ± 5.38 years (Figure 1).

**Figure 2:**
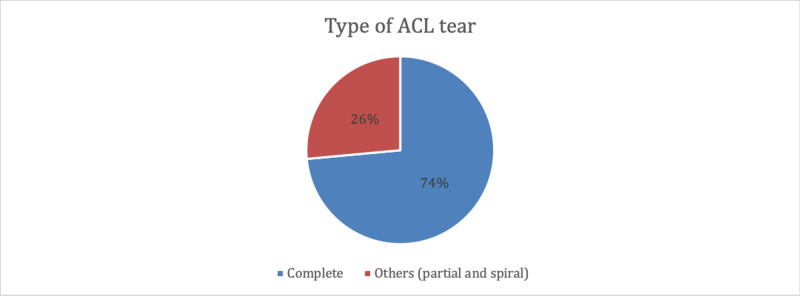
Type of anterior cruciate ligament (ACL) tear in the included patients with ACL injury

The ACL injury was located on the right side in approximately two-thirds of patients (62.5%). In the majority of patients n=100 (73.5%), the ACL tear was complete, and in the remaining (n=36, 26.5%), it was partial or spiral (Figure 2). 

**Figure 3:**
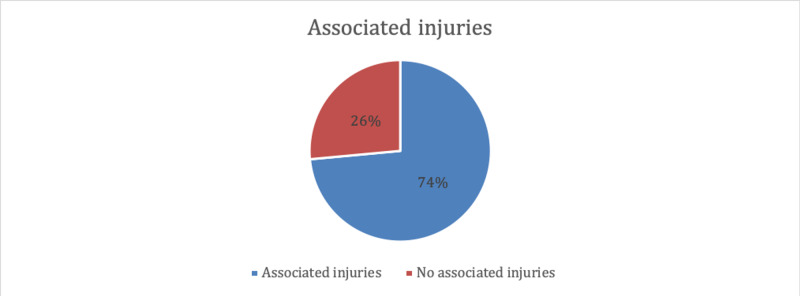
Associated injuries in the included patients with anterior cruciate ligament (ACL) injury

Associated injuries were found in the majority of patients (73.5%) (Figure 3). 

The medial meniscus was injured in 67 patients (49.3%), whereas the lateral meniscus was injured in 22 patients (16.2%). Regarding collateral ligament injuries, the medial collateral ligament was injured in six patients (4.4%), the lateral collateral ligament in four patients (2.6%), and both collateral ligaments in three patients (2.2%). Bone injuries in the included patients with ACL injury were bone edema (FLC, tibia, fibula or femur) in 32 patients (23.5%) and Segond fracture in nine patients (6.6%). The two women included were aged ≤25 years. There was a significant relationship between the age group and side of ACL injury (P<0.05) but not between the age group and presence of associated injury, type of ACL tear, meniscus injury, bone injury, or type of collateral ligament injury in all patients (P>0.05). Most patients were men (n=134, 98.5%) (Table 1).

**Table 1:** Relationship between the age group and side of anterior cruciate ligament (ACL) injury, associated injury, type of ACL tear, meniscus, bone injury, and collateral ligament in the included patients with ACL injury

		Age group (years)				Total (N=136)	
		≤ 25 (N=44)	26-30 (N=45)	31- 35 (N=26)	> 35 (N=21)		
Sex	Female	2	0	0	0	2	0.236
		4.5%	0%	0%	0%	1.5%	
	Male	42	45	26	21	134	
		95.5%	100.0%	100.0%	100.0%	98.5%	
Side of ACL injury	Left	26	18	6	1	51	0.001
		59.1%	40.0%	23.1%	4.8%	37.5%	
	Right	18	27	20	20	85	
		40.9%	60.0%	76.9%	95.2%	62.5%	
ACL tear	Complete	34	31	17	18	100	0.347
		77.3%	68.9%	65.4%	85.7%	73.5%	
	Other	10	14	9	3	36	
		22.7%	31.1%	34.6%	14.3%	26.5%	
Associated injury	No	16	12	4	4	36	0.217
		36.4%	26.7%	15.4%	19.0%	26.5%	
	Yes	28	33	22	17	100	
		63.6%	73.3%	84.6%	81.0%	73.5%	
Meniscus injury	Lateral	8	7	3	4	22	0.488
		18.2%	15.6%	11.5%	19.0%	16.2%	
	Medial	17	24	17	9	67	
		38.6%	53.3%	65.4%	42.9%	49.3%	
	None	19	14	6	8	47	
		43.2%	31.1%	23.1%	38.1%	34.6%	
Collateral ligament injury	Both	0	1	2	0	3	0.294
		0%	2.2%	7.7%	0%	2.2%	
	Lateral	2	1	1	0	4	
		4.5%	2.2%	3.8%	0%	2.9%	
	Medial	1	1	3	1	6	
		2.3%	2.2%	11.5%	4.8%	4.4%	
	None	41	42	20	20	123	
		93.2%	93.3%	76.9%	95.2%	90.4%	
Bone injury	Bone edema	9	8	9	6	32	0.430
		20.5%	17.8%	34.6%	28.6%	23.5%	
	Segond Fracture	2	2	3	2	9	
		4.5%	4.4%	11.5%	9.5%	6.6%	
	None	33	35	14	13	95	
		75.0%	77.8%	53.8%	61.9%	69.9%	

There was also a significant relationship between the type of ACL tear and side of ACL injury and presence of associated injury (P<0.05) but not between the type of ACL tear and age, sex, meniscus injury, bone injury, or type of collateral ligament injury in all patients (P>0.05) (Table 2). 

**Table 2:** Relationship between the type of anterior cruciate ligament (ACL) tear and age group, sex, side of ACL injury, associated injury, meniscus, bone injury, and collateral ligament in the included patients with ACL injury

		Type of ACL tear		Total (n=136)	P value
		Complete (n=100)	Others (n=6)		
Age group (years)	≤ 25	34	10	44	0.347
		34.0%	27.8%	32.4%	
	26-30	31	14	45	
		31.0%	38.9%	33.1%	
	31-35	17	9	26	
		17.0%	25.0%	19.1%	
	>35	18	3	21	
		18.0%	8.3%	15.4%	
Sex	Female	1	1	2	0.461
		1.0%	2.8%	1.5%	
	Male	99	35	134	
		99.0%	97.2%	98.5%	
Side of ACL injury	Left	37	14	51	0.017
		37.0%	38.9%	37.5%	
	Right	63	22	85	
		63.0%	61.1%	62.5%	
Bone injury	Bone edema	25	7	32	0.111
		25.0%	19.4%	23.5%	
	Segond Fracture	9	0	9	
		9.0%	0%	6.6%	
	None	66	29	95	
		66.0%	80.6%	69.9%	
Meniscus	Lateral	17	5	22	0.575
		17.0%	13.9%	16.2%	
	Medial	51	16	67	
		51.0%	44.4%	49.3%	
	None	32	15	47	
		32.0%	41.7%	34.6%	
Presence of associated injury	No	21	15	36	0.017
		21.0%	41.7%	26.5%	
	Yes	79	21	100	
		79.0%	58.3%	73.5%	
Collateral ligament injury	Both	2	1	3	0.944
		2.0%	2.8%	2.2%	
	Lateral	3	1	4	
		3.0%	2.8%	2.9%	
	Medial	5	1	6	
		5.0%	2.8%	4.4%	
	None	90	33	123	
		90.0%	91.7%	90.4%	

## Discussion

The study aimed to address the prevalence of ACL injuries and its association with different types of knee injuries in these patients. The majority of ACL injuries were on the right side (62.5%), whereas left-sided injures were less common (37.5%).

Similar to our results, in Arar City, Kingdom of Saudi Arabia, another cross-sectional study was conducted among a representative sample of Northern Border University students; right-sided injuries were reported in 60.6% and left-sided injuries in 39.4% of cases [14]. A retrospective cross-sectional study conducted among students of physical education at Umm Al-Qura University found that right-sided injures accounted for 70% of cases, whereas left-sided injuries accounted for 30% [10]. In addition, another study reported that right-sided injuries were the most common (56.9%) [15].

Regarding the type of ACL tear, it was complete in 73.5% of cases and partial or spiral in 26.5% of cases in our study. In agreement with our results, another study reported that the ACL tear was complete in 54.9% of cases and partial in 38% of them [14]. In Makkah, another study reported partial ACL injury in 23.7% of cases and complete ACL injury in 37.9% of cases [9]. Moreover, another study found complete injury in 81.3% of cases and partial injury in 12.7% of cases [16]. 

Associated injuries were found in the majority of cases (73.5%) in this study: bone edema (FLC, tibia, fibula, or femur) was reported in 23.5% of cases and Segond fracture in 6.6% of cases. In accordance with our findings, another study found that there was associated injury in 73.2% of cases [14]. Additionally, that study reported associated injuries of contusions of the knee in 50.7%, twisting of the knee in 22.5%, twisting of the foot in 11.3%, wounds in the knee in 8.5%, wounds and contusions on the body in 4.2%, fractured hand in 1.4%, and fractured femur in another 1.4% of cases [14]. The menisci play a crucial role in knee homeostasis, lubrication, joint stability, proprioception, and shock absorption [17]. ACL tears are frequently associated with meniscal injury. Numerous authors have described associations between the presence of a meniscal tear at the time of ACL reconstruction, time to surgery, and the number of instability episodes [18]. In our study, the medial meniscus was injured in 49.3% of cases, whereas the lateral meniscus was injured in 16.2%. Similar to our results, another study found that 44.4% of cases had meniscal tears, of which 30.5% were medial, 7.2% were lateral, and 6.7% had both lateral and medial meniscal tears [19]. In contrast, Millett et al. study found that meniscal injury occurred more frequently in the medial meniscus than in the lateral meniscus [20]. Similarly, another study reported lateral meniscus represented 41% of cases, whereas medial meniscus represented 25.6% of them [21].

Ghodadra et al. studied the incidence of medial and lateral meniscal tears in 709 patients with ACL tears and found that 37% had medial meniscal tears and 41% had lateral meniscal tears, whereas 17% had both lateral and medial meniscal tears [22]. Hagino et al. showed that the incidence of meniscal tear was 72.7% (256 patients) in acutely injured knees, with medial meniscal tears only found in 11% of patients, lateral meniscal tears in 69%, and both medial and lateral meniscal tears in 20% [23]. In this study, the incidence of medial and lateral meniscal tears was 49.3 and 16.2%, respectively. The total percentage of both meniscal tears thus correlates with the findings obtained from previous studies. However, we found that the medial meniscus is more commonly injured than the lateral meniscus. This appears to be due to the strong attachment of the medial meniscus to the joint capsule and the deep fibers of the medial collateral ligament, making it more stable than the lateral meniscus, which does not connect with the lateral collateral ligament and has looser attachments with the joint capsule. 

Regarding collateral ligament injuries, medial injury was reported in 4.4%, lateral injury in 2.9%, and bilateral injury in 2.2% of the cases in our study. In contrast to our results, another study reported that 4.5% of cases had collateral ligament injuries: the majority of them (2.4%) had injuries to both collateral ligaments, 1.4% had a medial collateral ligament injury, and 0.6% had a lateral collateral ligament injury only [24].

Our study found a significant relationship between the age group and side of ACL injury (P<0.05) but not between the age group and presence of associated injury, type of ACL tear, meniscus injury, bone injury, and type of collateral ligament injury in all patients with ACL injury (P>0.05).

There was a significant relationship between the type of ACL tear, side of ACL injury, and presence of associated injury (P<0.05) but not between the type of ACL tear and age, sex, meniscus injury, bone injury, or the type of collateral ligament injury in our patients (P>0.05). Regarding the relationship between bone injury and other injuries (meniscus and collateral ligaments), our study found a significant association with collateral ligament injury but not with meniscus injury.

Our study was limited by the small sample size and the fact that it was a single-center study. Although it would have been preferable to include all hospitals in Jeddah, our study investigated all injuries associated with ACL injury, which is very important.

## Conclusions

This study investigated the association between ACL and other associated knee injuries using MRI for diagnosis. After assessing many different factors, we found no significant relationship between ACL injuries and meniscal, collateral ligament, or bone injuries. We recommend further research in this particular field with a larger and more divergent sample size. Multi-center studies are needed in the future to further support the results of the present study.
